# Unusual Case of Peroneal Nerve Palsy Due to Compression of an Extraneural Intramuscular Synovial Cyst: A Case Report

**DOI:** 10.7759/cureus.110319

**Published:** 2026-06-05

**Authors:** Daniel Schrednitzki, Stephanie Schneider, Jonas Sina, Stefan Kroppenstedt, Andreas M Halder

**Affiliations:** 1 Orthopaedics, Traumatology, Hand- and Reconstructive Surgery, Sana Hospital Lichtenberg, Berlin, DEU; 2 Orthopaedic Surgery, Hand Surgery, and Traumatology, City Hospital Zurich, Zurich, CHE; 3 Neurosurgery, Sana Hospital Sommerfeld, Kremmen, DEU; 4 Orthopaedic Surgery, Sana Hospital Sommerfeld, Kremmen, DEU

**Keywords:** compression, intraneural cyst, neuropathy, peroneal nerve, synovial cyst

## Abstract

Peroneal neuropathy is the most common neuropathy in the lower limb, typically caused by external compression over the peroneal head due to poor soft tissue protection, while internal compression by cysts is unusual. We present a case of an unusual compression neuropathy of the deep peroneal nerve caused by an intramuscular extraneural synovial cyst with acute symptomatology. A 45-year-old male experienced a sudden onset of foot drop following knee distortion, with neurophysiological studies demonstrating severe axonal injury and MRI revealing a 7.8×2×2.3 cm multilobular cystic mass within the peroneus longus muscle originating from the tibioperoneal joint. Surgical exploration revealed the peroneal nerve with external damage changes through an intramuscular mucinous cyst, which was completely excised, including its stalk, with histological examination confirming a ganglion cyst without malignancy. Progressive recovery of sensibility and motor function occurred within five days postoperatively. Focal nerve entrapment of the peroneal nerve by a synovial cyst is not common but must be considered as a differential diagnosis, and in acute peripheral nerve neuropathies with severe dysfunction, immediate MRI is recommended to rule out compressive masses requiring surgical intervention, as delay in correct diagnosis can be catastrophic for nerve function prognosis.

## Introduction

Synovial cysts are relatively common lesions and are often asymptomatic, but they rarely cause neurological compression in the lower extremity [1]. The first case of peroneal nerve compression by a ganglion was reported by Sultan in 1921 [2]. Since then, only a limited number of reports have described ganglion-related neuropathies of the peroneal nerve [3-8], including lesions at the popliteal level, along the peroneus longus muscle [4-6], or within the nerve itself as intraneural ganglion cysts.

The common peroneal nerve arises from the sciatic nerve and wraps around the fibular neck in a superficial position, making it particularly susceptible to external compression and trauma. Compression at this site can lead to weakness of ankle dorsiflexion and foot eversion, resulting in foot drop and sensory disturbances over the lateral leg and dorsum of the foot. Ganglion cysts originating from the proximal tibiofibular joint or adjacent structures may compress the nerve either within the nerve sheath (intraneural) or from outside the nerve (extraneural), leading to acute or subacute peroneal neuropathy [1,3-7].

This case report describes an unusual presentation of acute peroneal nerve compression due to an extraneural intramuscular synovial cyst, with rapidly progressive foot drop. The combination of acute onset, severe neurological deficit, and the intramuscular location of the compressive lesion makes this case particularly relevant for clinical practice and for the differential diagnosis of peroneal nerve palsy.

## Case presentation

Patient demographics and initial presentation

A 45‑year‑old male patient felt sudden pain in his right knee after standing up from a kneeling position, which was caused by a twisting injury in his right knee. No direct trauma was reported. Subsequently, he described pain on the upper lateral side of the calf. During the following night, the patient noticed mild paresthesia in the lateral calf and toes along with loss of strength, especially in the hallux. The next day, he experienced a steppage gait consistent with foot drop.

Medical history and neurophysiological studies

The patient initially presented to an external physician shortly after symptom onset, but no specific diagnosis was made at that time. Two weeks after the incident, he underwent nerve conduction studies that showed small distal compound motor action potentials of the common peroneal nerve and a proximal amplitude of 0. Needle EMG demonstrated pathologic spontaneous activity without voluntary motor unit recruitment in peroneal‑innervated muscles, indicating a severe axonal injury to the common peroneal nerve at or below the level of the fibular head (Figure 1). Eight weeks later, repeat nerve conduction studies showed absent compound motor action potentials of the peroneal nerve. Needle EMG of the tibialis anterior and extensor hallucis longus demonstrated pathologic spontaneous activity with only minimal voluntary motor unit recruitment, indicating an almost complete peroneal nerve lesion without evidence of reinnervation potentials (Figure 2).

**Figure 1:**
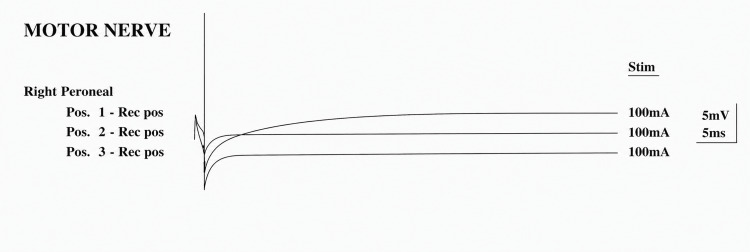
First neurophysiological study (week 2) demonstrating severe common peroneal axonal injury with markedly reduced distal compound motor action potentials and absent proximal amplitude (0 µV).

**Figure 2:**
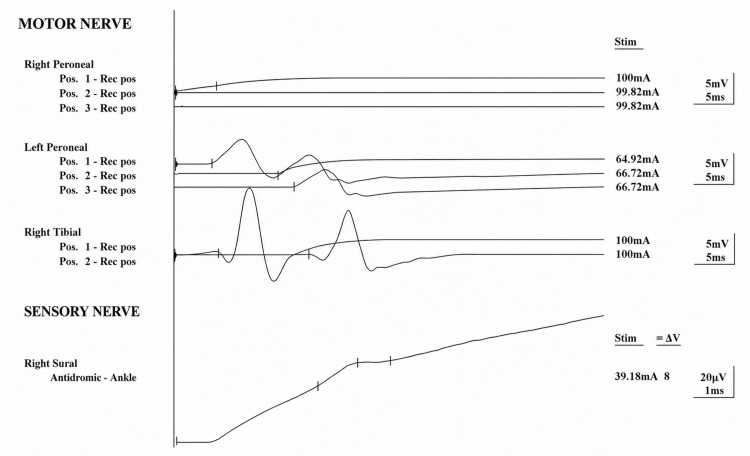
Neurography demonstrating a complete common peroneal nerve lesion with absent potential and amplitude (0 µV) and no signs of reinnervation.

Clinical examination at three months 

Three months after the beginning of symptoms, the patient was referred to our department, where we had our first contact and initiated further diagnostic workup and treatment. Physical examination showed atrophy of the peroneus muscles and the tibialis anterior muscle. Manual muscle testing using the Medical Research Council (MRC) scale revealed 0/5 strength in the tibialis anterior, extensor hallucis longus, and extensor digitorum muscles, with a drop foot and steppage gait.

Magnetic resonance imaging

On MRI, a hypodense, multilobular, 7.8×2×2.3 cm cystic mass was detected around the fibular neck, inside the M. peroneus longus, with an origin in the tibioperoneal joint and extending distally to the syndesmosis (Figure 3).

**Figure 3:**
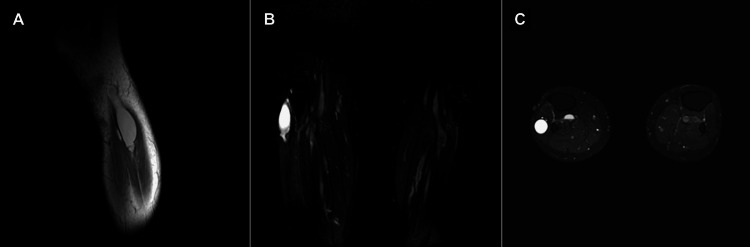
MRI of the right proximal lower leg demonstrating a multilobular cystic mass within the peroneus longus muscle arising from the proximal tibiofibular joint. On the T2-weighted coronal (A) and proton density-weighted sagittal (B) images, the lesion appears homogeneously hyperintense with a thin wall, consistent with a synovial or ganglion cyst. The axial T2‑weighted image (C) shows the close spatial relationship between the cyst and the common peroneal nerve at the fibular neck.

Surgical intervention

The surgical exploration revealed the peroneal nerve with external changes of damage through abnormal color (Figure 4). No intraneural lesions or masses were found. Thereafter, we opened the fascia of the peroneus longus muscle and found an intramuscular mucinous cyst (Figure 5). The cyst size was approximately 8×3×2 cm and was not in direct contact with the peroneal nerve (Figure 6). We determined that the origin of the cyst was in the tibioperoneal joint. The cyst was completely excised with its stalk and base.

**Figure 4:**
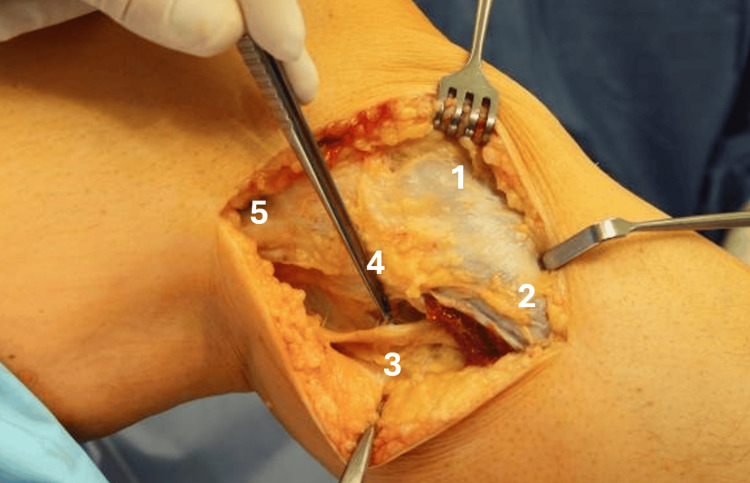
Intraoperative view of the right lateral knee showing the common peroneal nerve at the fibular neck. The nerve appears flattened and slightly discolored along its course over the fibular head, compatible with chronic external compression by the adjacent intramuscular cyst. Intramuscular ganglion cyst (1) within the peroneus longus muscle (2), common peroneal nerve (3) at the level of the fibular head (4), and the tendon of the biceps femoris muscle (5).

**Figure 5:**
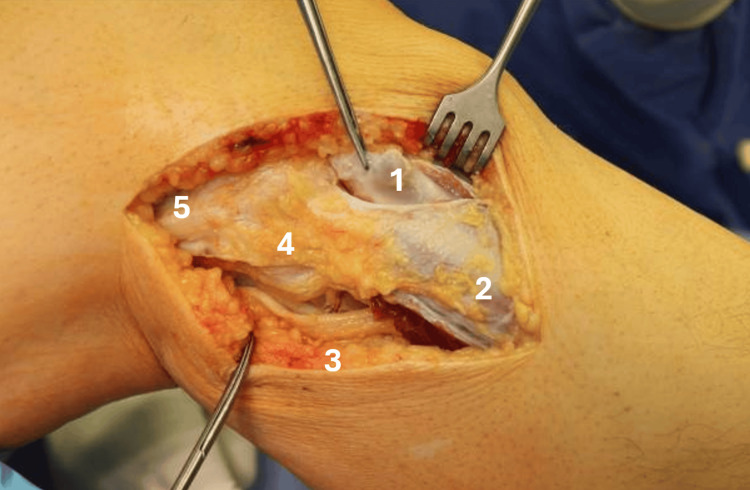
The fascia of the peroneus longus muscle was opened to reveal an intramuscular ganglion cyst in the proximal lower leg. Intramuscular ganglion cyst (1), peroneus longus muscle (2), common peroneal nerve (3) at the level of the fibular head (4), and biceps femoris tendon (5).

**Figure 6:**
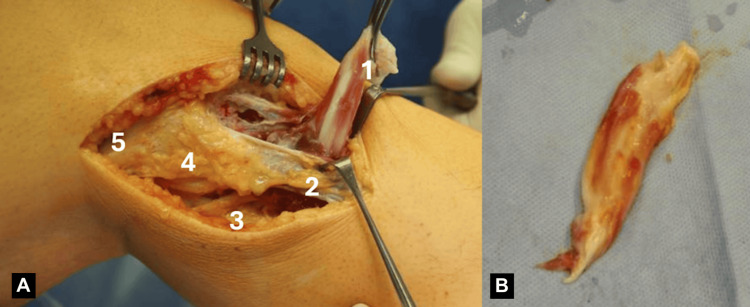
Complete excision of the intramuscular ganglion cyst. Intraoperative view (A) showing the cyst being mobilized from within the peroneus longus muscle after detachment from its stalk at the proximal tibiofibular joint. The specimen on the back table (B) demonstrates the elongated multilobular cyst with its fibrous stalk and articular branch connection. Intramuscular ganglion cyst (1) within the peroneus longus muscle (2), common peroneal nerve (3) at the level of the fibular head (4), and biceps femoris tendon (5).

Histopathology 

Histological examination revealed the presence of a fibrous wall cyst, consistent with a ganglion. No evidence of malignancy was found. 

Postoperative course

During the next five days, the patient noticed progressive recovery of sensibility, complete resolution of pain, and a clear improvement in active foot motion. On neurological examination, muscle strength had improved to 3/5 in the tibialis anterior and extensor digitorum muscles and 1/5 in the extensor hallucis longus. Given the later electrodiagnostic findings of an almost complete peroneal nerve lesion without reinnervation potentials at week 8, we interpret this early postoperative improvement as recovery from conduction block and relief of nerve compression rather than as true axonal regeneration.

Ethics and informed consent

Written informed consent was obtained from the patient for publication of this case report and the accompanying images. The case description was prepared in accordance with the principles of the Declaration of Helsinki. According to the policies of our institution, formal approval from an ethics committee was not required for a single anonymized case report.

## Discussion

Pathophysiology and articular theory

Ganglion cysts are benign lesions, containing mucinous fluid, rich in hyaluronic acid ​[6]​. The pathogenesis of ganglion cysts has been increasingly understood through the articular (synovial) theory, which posits that intraneural ganglion cysts develop from synovial joints through capsular defects ​[7,8]​. Joint fluid egresses through a rent in the joint capsule and courses along an articular nerve branch into the parent nerve, following the path of least resistance ​[7]​. This theory has gained substantial support from recent case series and is now widely accepted in clinical practice ​[8,9]​.

In the lower limb, the most common compression neuropathy affects the peroneal nerve. The main cause of peroneal palsy is external compression over the peroneal head ​[10,11]​ because it has poor soft tissue protection (cross-leg positions, casts, knee protectors, direct trauma). Internal compression caused by cysts is less common but remains an important consideration in the differential diagnosis [12].

Limitations

A major limitation of this case is the short follow‑up period of only five days after surgery. Although the early postoperative course was favorable, no further objective assessments of motor function or potential recurrence were available, as the patient did not attend subsequent scheduled follow‑up visits. This restricts our ability to comment on long‑term outcomes.

Anatomical considerations and cyst classification

Ganglion cysts have been reported to be present in a variety of locations, including the substance of the nerve itself (intraneural) and within surrounding tissues such as the peroneus longus muscle (extraneural) ​[10,13]​. Two forms of compression are recognized: intraneural cysts that infiltrate and fuse with nervous tissue and extraneural cysts that exert compressive action without infiltrating the nerve ​[13,14]​. In cases of intraneural distribution, the cyst usually arises from the proximal tibiofibular joint and reaches the common peroneal nerve via the articular recurrent branch ​[7,15]​.

Recent literature has documented various locations for peroneal nerve compression by ganglion cysts, including the supracondylar area of the femur ​[16]​, the popliteal fossa ​[17]​, and the proximal tibiofibular joint ​[13,14]​. While most reported cases involve intraneural ganglion cysts, extraneural variants remain rare, particularly those located intramuscularly, as in our case ​[16]​.

Clinical presentation and acute symptomatology

The ganglion can be asymptomatic. When symptoms appear, they are typically progressive and intermittent due to the cyclic and triphasic course (growth, plateau, and diminution stages) [4]. This progressive pattern is well-documented in recent case series [13,18].

However, acute symptoms, as presented in our case, are uncommon. We consider that our patient developed an acute growth (due to synovial swelling) of a pre-existing synovial cyst or weakness of the synovial membrane of the joint caused by indirect trauma (distortion of his right knee). The acute growth of the cyst resulted in rapid nerve compression and neurological impairment. Recent reports have documented similar acute presentations, though they remain rare ​[17,18]​.

Literature comparison and clinical patterns

Clinical findings in peroneal nerve compression by ganglion cyst were described by Stack et al. ​[19]​ in nine cases. Patients complained of pain in the majority of cases; three cases referred to a palpable mass in the area of the upper fibula, one patient presented a foot drop, and surprisingly, no sensory abnormalities were reported.

Muckart [5]​ reported eight cases of intramuscular ganglion. Of these cases, five patients complained of pain, seven had swelling on the lateral side of the calf, five presented neurological motor findings, and two of them also reported sensitive symptoms (anesthesia in the first interdigital cleft). Motor function is more affected than sensory function, and the extensor hallucis longus is the most severely affected muscle and the last for recovery, as confirmed in our case and supported by recent studies ​[18,20]​.

Diagnostic approach

In addition to clinical examination, electrophysiological testing is crucial for localizing the lesion and evaluating disease progression, although it does not reveal the underlying cause [9,10,20]. EMG helps differentiate peroneal neuropathy from L5 radiculopathy and other peripheral nerve lesions [20].

MRI offers the most useful method to detect and assess peripheral nerve pathologies [19,21]. It can identify compressive causes, clarify differential diagnoses, and reveal the relationship between the cyst and surrounding anatomical structures, including its connection to the joint through a pedicle [13,16]. Recent literature emphasizes that ultrasonography can serve as an effective screening tool due to its portability and cost-effectiveness, though MRI remains superior for surgical planning [16,17,22].

Treatment and surgical outcomes

Treatment for symptomatic ganglions is surgical excision. Complete removal of the cyst, including the stalk and its base in the joint, is essential because incomplete excision can lead to recurrence ​[1,4,13]​. Recent surgical series have demonstrated that careful dissection using microscopic techniques often permits total removal of intraneural cysts without damage to nerve fascicles ​[14,18]​.

Contemporary studies report favorable surgical outcomes with low recurrence rates when complete excision, including the articular branch connection, is performed [8,9,23]. A systematic review of 128 patients treated surgically demonstrated improved motor function in all cases, with recurrence rates varying from 0% to 25%, most being extraneural recurrences ​[23]​. Recovery typically occurs within days to weeks after operative decompression, with median postoperative muscle strength improving to MRC grade 4-5 ​[18,23,24]​. However, the timing of intervention is critical, as a delayed diagnosis can result in irreversible nerve damage [16,17].

In our case, the rapid postoperative improvement within five days aligns with recent reports emphasizing that early surgical intervention before significant axonal degeneration leads to optimal functional recovery ​[16]​.

Clinical implications

Recent consensus in the literature supports immediate surgical excision when neurological symptoms are present, rather than observation or conservative management with aspiration ​[13,14,23]​. The role of complete pedicle resection and, in some cases, resection of the superior tibiofibular joint has been increasingly emphasized to prevent recurrence ​[10,25]​. Follow-up imaging is recommended to detect potential recurrence, which may occur in 5-25% of cases despite adequate initial surgery ​[13,23]​.

## Conclusions

Focal entrapment of the common peroneal nerve by a synovial cyst is uncommon but should be considered in the differential diagnosis of patients presenting with acute or progressive lower extremity weakness and foot drop. Although spontaneous recovery may occur in some cases of external compression neuropraxia, early imaging - preferably MRI when available - should be strongly considered in cases of severe acute dysfunction to rule out compressive masses that may require surgical intervention, as delay in diagnosis can be detrimental to nerve function. In our patient, timely surgical decompression with complete cyst excision, including the stalk and articular branch connection, was associated with clear early motor improvement at five days postoperatively; however, longer follow‑up would be necessary to fully assess long‑term recovery and the risk of recurrence. Extraneural intramuscular cysts should be included in the differential diagnosis when neurophysiology localizes the lesion to the fibular neck but no intraneural pathology is identified.
